# A Multi-Fidelity Data Fusion Approach Based on Semi-Supervised Learning for Image Super-Resolution in Data-Scarce Scenarios

**DOI:** 10.3390/s25175373

**Published:** 2025-08-31

**Authors:** Hongzheng Zhu, Yingjuan Zhao, Ximing Qiao, Jinshuo Zhang, Jingnan Ma, Sheng Tong

**Affiliations:** 1 Air Traffic Control and Navigation School, Air Force Engineering University, Xi’an 710051, China; zhuhongzheng803@163.com (H.Z.); fragileleee@163.com (X.Q.); 17594470769@163.com (J.Z.); 2 Fundamentals Department, Air Force Engineering University, Xi’an 710051, China; feixu0924@163.com

**Keywords:** semi-supervised learning, image reconstruction, multi-fidelity data fusion

## Abstract

Image super-resolution (SR) techniques can significantly enhance visual quality and information density. However, existing methods often rely on large amounts of paired low- and high-resolution (LR-HR) data, which limits their generalization and robustness when faced with data scarcity, distribution inconsistencies, and missing high-frequency details. To tackle the challenges of image reconstruction in data-scarce scenarios, this paper proposes a semi-supervised learning-driven multi-fidelity fusion (SSLMF) method, which integrates multi-fidelity data fusion (MFDF) and semi-supervised learning (SSL) to reduce reliance on high-fidelity data. More specifically, (1) an MFDF strategy is employed to leverage low-fidelity data for global structural constraints, enhancing information compensation; (2) an SSL mechanism is introduced to reduce data dependence by using only a small amount of labeled HR samples along with a large quantity of unlabeled multi-fidelity data. This framework significantly improves data efficiency and reconstruction quality. We first validate the reconstruction accuracy of SSLMF on benchmark functions and then apply it to image reconstruction tasks. The results demonstrate that SSLMF can effectively model both linear and nonlinear relationships among multi-fidelity data, maintaining high performance even with limited high-fidelity samples. Finally, its cross-disciplinary potential is illustrated through an audio restoration case study, offering a novel solution for efficient image reconstruction, especially in data-scarce scenarios where high-fidelity samples are limited.

## 1. Introduction

In modern image processing and computer vision tasks, image reconstruction techniques, particularly image super-resolution reconstruction (SR), have emerged as a fundamental and widely applied research direction. SR technology aims to recover high-resolution (HR) images from one or multiple low-resolution (LR) inputs, thereby enhancing visual quality and information density [1]. Its applications span diverse domains, including medical image reconstruction [2,3], satellite remote sensing image analysis [4,5], and flow field reconstruction [6,7], demonstrating that it holds significant value for improving image interpretation accuracy and downstream task performance. Consequently, the advancement of image super-resolution technology represents an inevitable trend in the evolution of image processing methodologies and a critical interdisciplinary bridge for industrial implementation.

Current image super-resolution reconstruction methods are primarily categorized into interpolation-, reconstruction-, and learning-based approaches [8]. Traditional interpolation algorithms (e.g., nearest-neighbor, bilinear, and bicubic interpolation) serve as the foundation for image reconstruction due to their computational efficiency, ease of implementation, and capacity to preserve global structures. Bilinear interpolation achieves smooth transitions through weighted averaging of neighboring pixels, effectively reducing aliasing effects [9]. Bicubic interpolation employs cubic polynomial fitting of neighboring pixels, significantly enhancing edge sharpness [10]. In scenarios with high real-time requirements (e.g., video streaming), interpolation algorithms are preferred for their low computational complexity (e.g., nearest-neighbor interpolation has a complexity of O(1)). However, these methods exhibit multiple fundamental limitations. First, due to the inherent low-pass characteristics of interpolation kernels, they struggle to effectively preserve high-frequency details in images, leading to significant degradation of texture and edge information, particularly in complex nonlinear structures (e.g., fine fabric patterns), where blurring is more pronounced [11]. Second, interpolation methods often introduce noticeable artificial artifacts. For instance, nearest-neighbor interpolation tends to produce jagged edges and mosaic effects, bilinear interpolation creates blocky distortions under high magnification [12], and bicubic interpolation may result in excessive smoothing or oscillatory artifacts [13]. Finally, these methods face a trade-off between computational efficiency and reconstruction quality: higher-order methods incur substantial computational overhead and poor real-time performance, while lower-order methods are fast but lack precision. Similarly, traditional interpolation methods face several limitations, they often rely on strong assumptions about the smoothness and stationarity of the underlying functions, which may not hold in complex real-world problems. Their performance deteriorates significantly in high-dimensional parameter spaces due to the curse of dimensionality. They are less flexible in capturing highly nonlinear or heterogeneous fidelity gaps across domains.

Image reconstruction methods based on sparse representation and edge priors introduce certain structural modeling capabilities, yet their reconstruction quality remains constrained by prior design. To address this, Yang et al. [14] proposed a joint training approach for low- and high-resolution image patch dictionaries, leveraging sparse representation of image patches under an overcomplete dictionary to achieve high-resolution reconstruction. This method demonstrates superior performance in preserving local structures and restoring fine details while exhibiting certain noise robustness, making it a representative application of sparse modeling in image reconstruction. However, its ability to model edge structures is limited, often leading to block artifacts, and it suffers from high computational costs in both dictionary training and inference. To further enhance reconstruction performance, Zhang et al. [15] introduced an adaptive sparse representation strategy combined with joint dictionary training. By accounting for the diversity of local image structures, their method adaptively selects the optimal sparse basis for each low-resolution patch while jointly training low- and high-resolution dictionaries to maintain consistent sparse coefficients, thereby achieving more refined reconstruction results. This approach improves the representational capacity across image patches and effectively enhances reconstruction quality. Nevertheless, it still faces computational efficiency challenges and relies on dictionary training.

## 2. Related Work

In recent years, with the rapid advancement of deep learning techniques, deep neural network-based image super-resolution reconstruction methods have emerged as a mainstream research direction. Leveraging the powerful feature extraction and nonlinear mapping capabilities of deep network architectures, these methods can maintain semantic consistency in pixel-level reconstruction while significantly improving reconstruction quality in complex scenarios. Early approaches, such as the super-resolution convolutional neural network (SRCNN) proposed by Dong et al. [16], achieved end-to-end mapping between low- and high-resolution images via convolutional neural networks. This lightweight structure offered fast processing speeds and pioneered the application of deep learning in super-resolution. However, it relied on bicubic interpolation for preprocessing, which could amplify noise and introduce blurring, while its generalization ability in complex scenes remained limited. To overcome the limitations of traditional interpolation and sparse coding methods in detail recovery and computational efficiency, Zhang et al. [17] proposed a sparse reconstruction strategy combining a non-subsampled contourlet transform and Gabor feature extraction. They further constructed a multi-residual fusion structure (MRMFSCSR) based on an improved very deep super-resolution (VDSR) network, enhancing reconstruction quality while improving high-frequency detail recovery. However, this came at the cost of increased model complexity. Further advancements were made by Yan et al. [18], who introduced multi-layer convolutional structures and residual learning mechanisms. By incorporating sub-pixel convolutional layers, they significantly improved texture reconstruction while enhancing training efficiency and reconstruction performance. Addressing the scarcity of high-resolution ground truth images, Mishra et al. [19] proposed the unsupervised method called Self-FuseNet, which leverages a UNet architecture to fuse multi-scale information from the image itself, enabling robust reconstruction without paired training data and demonstrating superior generalization ability. From the network architecture design perspective, Chen et al. [20] introduced a lightweight multi-level feature fusion network (MFFN) that enhances feature extraction efficiency through nested residual blocks and asymmetric channel compression mechanisms. This approach maintains high reconstruction accuracy even under parameter constraints, making it suitable for resource-limited scenarios. For medical images with blurred boundaries and pixel uncertainty, Wang et al. [21] incorporated fuzzy logic and attention mechanisms to produce a fuzzy hierarchical fusion convolutional neural network. This improved boundary delineation and multi-scale feature fusion, achieving outstanding performance on the COVID-CT dataset. Generative adversarial networks (GANs) have also been introduced to super-resolution tasks due to their strong generative capabilities. Deng et al. [22] proposed a GAN-based turbulence velocity field reconstruction framework that demonstrated excellent multi-scale feature recovery. However, GAN-based methods suffer from complex training processes, high computational resource consumption, and challenges in stability and hyperparameter sensitivity.

The above deep learning-based methods have made remarkable progress in image reconstruction, enhancing reconstruction accuracy and improving detail restoration capabilities. However, they still face numerous challenges and limitations in practical applications. First, these methods generally rely on high-quality, strictly aligned low-resolution and high-resolution (LR-HR) paired datasets. In specialized domains such as medical imaging, remote sensing, and complex flow field visualization, acquiring high-resolution images is extremely costly, and data annotation is challenging, resulting in a severe scarcity of high-quality training samples. Second, high-resolution images are often scarce in real-world scenarios, while their low-resolution counterparts are relatively abundant. This data imbalance exacerbates the difficulty of feature extraction during training. Moreover, the degradation process of low-resolution images typically leads to a significant loss of high-frequency information, making it difficult for reconstruction models to accurately restore fine details and edge structures. This ultimately results in blurry or distorted outputs. Furthermore, most existing methods assume that training and testing data follow the same distribution—an assumption that rarely holds in real-world settings—thereby limiting the model’s generalization ability. Additionally, the inherent "black-box" nature of deep neural networks reduces their interpretability and robustness when dealing with complex heterogeneous data. In particular, the reconstruction quality degrades significantly when facing varying signal-to-noise ratios or data sources. These challenges collectively hinder the broader adoption and application of deep learning-based methods in high-resolution image reconstruction tasks.

In this context, the multi-fidelity data fusion (MFDF) strategy is an effective approach to address the challenges above. This technique enhances the overall model’s learning efficacy and robustness by effectively integrating data from diverse sources, varying sampling resolutions, or different imaging conditions. By leveraging low-fidelity samples while preserving data diversity, MFDF strengthens the expressive capacity of high-fidelity information. Taking remote sensing imagery as an example, data acquired from different satellites, sensors, or time periods exhibit significant variations in resolution and quality. Traditional methods often treat such heterogeneous data as noise sources and discard them. In contrast, MFDF methods aim to extract latent complementary features and compensatory information from these datasets, thereby optimizing reconstruction quality.

Notably, mainstream multi-fidelity fusion methods predominantly rely on supervised learning frameworks, which still require substantial labeled HR images for training and thus fail to completely overcome the “high-quality data dependence” issue. Consequently, achieving effective data fusion and image reconstruction under data-scarce or even partially labeled conditions has become a critical challenge in this field. In recent years, semi-supervised learning methods have demonstrated impressive performance in natural language processing, speech recognition, and image classification tasks. Existing SSL approaches can be broadly categorized into generative methods [23,24,25,26,27], consistency regularization methods [28,29,30,31], graph-based methods [32,33,34], hybrid methods [35], and pseudo-labeling methods [36]. These methods leverage a large number of unlabeled samples in conjunction with a small set of labeled data to participate jointly in the training process. This enables models to achieve stronger generalization capabilities in data distribution modeling while reducing the reliance on expensive labeled data, offering good practical feasibility in engineering applications. Introducing SSL principles into the multi-fidelity data fusion process to construct novel model architectures tailored for image reconstruction tasks can improve data utilization efficiency and strengthen the model’s cross-domain understanding of samples with varying fidelity levels. This approach effectively mitigates the performance degradation of existing SR methods under data-deficient conditions. Emerging studies have begun exploring the collaborative modeling of multi-scale, multi-view, and spectral–spatial multimodal information in images. For instance, some approaches use image content as supervision signals while leveraging structural information as auxiliary inputs to guide reconstruction networks in jointly learning content and edge features [37]. Others employ teacher–student frameworks to generate pseudo-labels for unlabeled samples and conduct consistency training, progressively enhancing the model’s feature extraction capability [38].

Motivated by the above considerations, this paper proposes a semi-supervised learning-based multi-fidelity image reconstruction method (SSLMF) to overcome the performance bottleneck of existing models in scenarios with scarce high-fidelity data. We construct a unified deep learning framework with strong feature fusion capabilities and high reconstruction accuracy by incorporating structural guidance from multi-fidelity samples and self-supervised optimization strategies. SSLMF is specifically designed for image reconstruction in data-scarce scenarios, where it leverages low- and medium-fidelity data as supplements to scarce high-fidelity samples through semi-supervised learning, thereby reducing reliance on expensive labeled HR data. The proposed framework integrates image data from multiple fidelity levels, enabling the complementary and synergistic integration of feature information across different fidelity domains. This approach effectively mitigates information loss during high-dimensional feature reconstruction and significantly enhances the structural consistency and detail restoration capability of reconstructed images. Our study further demonstrates that coarse contour information from low-fidelity images can provide stable global structural constraints for high-fidelity image reconstruction. Therefore, incorporating data from three or more fidelity levels for fusion modeling is crucial for improving the generalization ability and practical applicability of super-resolution models. In summary, this work organically combines image reconstruction, multi-fidelity data fusion, and semi-supervised learning to propose a novel image super-resolution framework tailored for real-world applications. It provides new research perspectives and technical pathways for multi-source image understanding and reconstruction.

### Our Contributions

Overall, we make the following three original contributions:

- Novel formulation of fidelity as a pseudo-labeling task: We reformulate multi-fidelity modeling as a semi-supervised learning problem, where different fidelity levels are treated as latent labels. This formulation enables the model to perform fidelity-consistent learning across labeled and unlabeled data, which is not addressed by previous multi-fidelity learning methods.
- Unified fusion framework across fidelities and supervision levels: The SSLMF method introduces a collaborative learning strategy that integrates a small number of high-fidelity samples with a large amount of medium- and low-fidelity data, allowing the model to leverage both cross-fidelity and cross-supervision consistency to enhance reconstruction performance.
- Application to high-resolution image reconstruction with reduced cost: By exploiting low-cost and low-fidelity image data, SSLMF achieves high-precision reconstruction with significantly reduced reliance on expensive high-fidelity samples. This makes the method particularly valuable for practical deployment in high-resolution image processing scenarios where data labeling or acquisition is limited.

The structure of this paper is as follows. Section 3 introduces the proposed SSLMF method and analyzes the correlations among multi-fidelity data. In Section 4, the prediction accuracy of the one-dimensional benchmark function is experimentally validated. Moreover, the high-resolution reconstruction of images serves as a compelling demonstration of the method’s efficacy within the realm of data fusion. Section 5 summarizes the work described in this paper.

## 3. Methods

### 3.1. Correlations Among Multi-Fidelity Data

A key issue in multi-fidelity data modeling is determining the relationships between high- and low-fidelity data. The comprehensive correlations between data of different fidelities [39] are mainly expressed as

(1) $$y_{h}=\lambda_{1}\left(x\right)y_{l}+\beta_{1}\left(x\right),$$

(2) $$y_{h}=\lambda_{2}\left(x\right)y_{m}+\beta_{2}\left(x\right),$$

and

(3) $$y_{m}=\lambda_{3}\left(x\right)y_{l}+\beta_{3}\left(x\right),$$

where $y_{l}$, $y_{m}$, and $y_{h}$ denote low-, medium-, and high-fidelity data, respectively. $\lambda_{1}\left(x\right)$ and $\beta_{1}\left(x\right)$, $\lambda_{2}\left(x\right)$ and $\beta_{2}\left(x\right)$, $\lambda_{3}\left(x\right)$ and $\beta_{3}\left(x\right)$ are the multiplication-related alternatives and the addition-related alternatives in the relationships between low- and high-fidelity data, between medium- and high-fidelity data, and between low- and medium-fidelity data, respectively. These expressions represent the linear relationships among the three data types, but nonlinear relationships exist in addition to these linear correlations. To better capture the nonlinear correlations during modeling, the above equations can be expressed as follows:

(4) $$y_{h}=F_{1}\left(x,y_{l}\right),$$

(5) $$y_{h}=F_{2}\left(x,y_{m}\right),$$

and

(6) $$y_{m}=F_{3}\left(x,y_{l}\right),$$

where $F_{j}\left(\cdot \right),\left(j=1,2,3\right)$ denotes a function mapping data of one fidelity to those of another fidelity. To adaptively represent linear or nonlinear relationships, $F_{j}\left(\cdot \right),\left(j=1,2,3\right)$ can be decomposed into linear and nonlinear parts as follows:

(7) $$F_{j}=\rho F_{jL}+\left(1-\rho \right)F_{jNL},\left(j=1,2,3\right),$$

where $F_{jL},F_{jNL}$ denote the linear and nonlinear terms in $F_{j}\left(\cdot \right),\left(j=1,2,3\right)$, respectively [40]. ρ is the weight hyperparameter between the linear and nonlinear data, which is automatically updated according to the data characteristics.

### 3.2. Model Construction

#### 3.2.1. Semi-Supervised Learning for Generating Pseudo-Labels

In multi-fidelity data fusion modeling, high-fidelity data are used as labeled data for model training. However, the difficulty and high cost of obtaining high-fidelity data mean that there are insufficient amounts of such data; in contrast, low-fidelity data are easy to obtain and exist in large quantities. Therefore, a problem of imbalanced data volumes occurs during model training, resulting in relatively poor training and insufficient accuracy. Semi-supervised learning [41] can be used to learn large volumes of data without requiring a large amount of labeled data. The labeled dataset is expanded by generating pseudo-labels. Semi-supervised learning has achieved outstanding results in the fields of image processing [42,43], natural language processing [44,45], and speech recognition [46], and has recently become a popular research direction in deep learning [47].

To date, semi-supervised learning has not been applied to data fusion tasks. This section analyzes the imbalance of data volumes in multi-fidelity data fusion. Typically, there is less high-fidelity labeled data but more low-fidelity data. We aim to use low-fidelity data corresponding to a small amount of high-fidelity data as labeled data and other low-fidelity data as unlabeled data for semi-supervised training.

**Figure 1:**
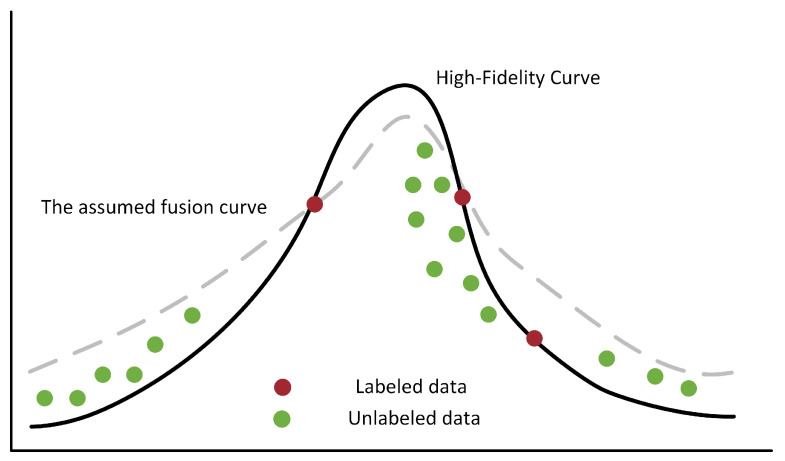
The benefits of using unlabeled data in semi-supervised learning for data fusion. The black solid line is a high-fidelity curve, and the dotted line is a hypothetical fusion curve. The red dots represent labeled data, and the green dots denote unlabeled data.

In semi-supervised learning, a prerequisite for the unlabeled data is that the samples used in the training process provide useful information for the labeled data [48]. This enables more information to be learned from unlabeled data, resulting in a robust model. The essence of data fusion is to map the low-fidelity data curve to the high-fidelity curve. If only a small amount of labeled data is available, we may obtain a hypothetical fusion rather than a correctly mapped high-fidelity data curve, because there will be fewer data points and insufficient information on the distribution trend. When using a large amount of unlabeled data, semi-supervised learning can extract more information so that the model produces a better mapping to the correct curve, as shown in Figure 1. The black solid line (High-fidelity curve) in the figure represents the ideal high-fidelity data curve, and the gray dashed line (Assumed fusion curve) is the hypothetical fusion result obtained when only a small number of red-labeled data points are used. It can be seen that there is a significant deviation from the real curve. When a large number of green unlabeled data points are introduced, the model can more accurately capture the overall trend of data distribution, making the fusion curve approach the high-fidelity curve.

**Figure 2:**
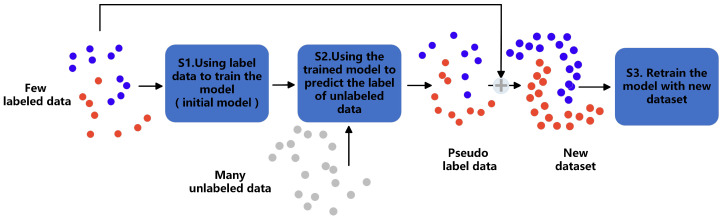
The semi-supervised learning process. The low-fidelity data corresponding to the high-fidelity data are used as labeled data, and the low-fidelity data without corresponding high-fidelity data are used as unlabeled data.

In the process of data fusion, the large amount of low-fidelity data provides a better representation of the distribution trend. These low-fidelity data are used as unlabeled data to provide information beneficial to model training. The proposed method uses semi-supervised learning to generate pseudo-labels for model training. Using high-fidelity data as labels, semi-supervised learning generates pseudo-labels for unlabeled data. The pseudo-labels and labeled data are used to form a new labeled dataset. The new labeled dataset provides additional distribution trend information for model training. In semi-supervised learning, pseudo-label noise and low confidence can substantially degrade reconstruction performance. Specifically, incorrect pseudo-labels may misguide the model into learning inaccurate feature representations, thereby introducing bias or ambiguity into the reconstruction task. High-noise pseudo-labels may cause the model to overfit incorrect samples, disrupting the potential consistency of data distribution. However, the weak supervised signals provided by low-confidence pseudo-labels may not be able to effectively restrain the reconstruction process, resulting in the loss of details or structural distortion. Therefore, to better apply the semi-supervised learning method of generating pseudo-labels to the data fusion task, all pseudo-labels generated in each round of training are added to the labeled dataset. Through iterative training, the pseudo-label data are updated after each round of training, and the model is continuously updated until convergence. This process is demonstrated in Figure 2. The core lies in gradually enhancing the utilization efficiency of low-fidelity data through a pseudo-label mechanism. Specifically, in the initial stage (S1), only a small amount of labeled data (red/blue dots) is used to train the initial model. At this time, the model’s understanding of the data distribution is limited, making it difficult to accurately capture the overall trend. Subsequently (S2), the initial model generates pseudo-labels for the unlabeled data (gray dots). Although these labels contain noise, they compensate for the sparsity of the labeled samples through a large amount of data, initially expanding the model’s understanding of the distribution trend. Finally (S3), the pseudo-labeled data is mixed with the original labeled data and the model is retrained to form a ’predictive-correction’ positive feedback loop.

The semi-supervised training process proceeds as follows. Let *X* be the input space and *Y* be the output space. The labeled dataset $D_{l}=\left\{\left(x_{1},y_{1}\right),\left(x_{2},y_{2}\right),\cdots ,\left(x_{n},y_{n}\right)\right\}$, where $x_{i}\in X$, $y_{i}\in Y$, and *n* is the number of labeled data. The unlabeled dataset $D_{u}=\left\{x_{n+1},x_{n+2},\cdots ,x_{n+m}\right\}$, where $x_{j}\in X$, and *m* is the number of unlabeled data. Suppose the model is $f_{\theta }:X\to Y$, where θ is the model parameter.

(1) Initial training based on labeled data. The loss function $L_{init}\left(\theta \right)$ is defined as the mean square error loss function, and the ReLU activation function is applied:

(8) $$L_{init}\left(\theta \right)=\frac{1}{n}\sum_{i=1}^{n}{\left(y_{i}-f_{\theta }\left(x_{i}\right)\right)}^{2}.$$

Here $y_{j}$ is the true value of the labeled data and $f_{\theta }\left(x_{j}\right)$ is the predicted value of the model for the input $x_{j}$. The parameter θ is updated using stochastic gradient descent: First, the gradient of $L_{init}\left(\theta \right)$ with respect to θ is found. Let $z_{i}={\left(y_{i}-f_{\theta }\left(x_{i}\right)\right)}^{2}$. Then the gradient is

(9) $$\frac{\partial L_{init}\left(\theta \right)}{\partial \theta }=\frac{1}{n}\sum_{i=1}^{n}\cdot \frac{\partial z_{i}}{\partial \theta }.$$

When

(10) $$z_{i}={\left(y_{i}-f_{\theta }\left(x_{i}\right)\right)}^{2}>0,$$

(11) $$\frac{\partial L_{init}\left(\theta \right)}{\partial \theta }=\frac{2}{n}\sum_{i=1}^{n}\left(y_{i}-f_{\theta }\left(x_{i}\right)\right)\cdot \frac{\partial f_{\theta }\left(x_{i}\right)}{\partial \theta }.$$

The model update rule is $\theta =\theta -\alpha \nabla_{\theta }L_{init}\left(\theta \right)$, where α is the learning rate.

(2) Generation of pseudo-labels. For unlabeled data, $x_{j}\in D_{u}$: $f_{\theta }$ is predicted through the trained model to obtain the prediction result $p_{j}=f_{\theta }\left(x_{j}\right)$. Since the model contains the ReLU activation function, the activation function acts during the prediction process. A pseudo-label is generated: ${\hat{y}}_{j}=p_{j}$.

(3) Training with labeled and pseudo-labeled data. A new dataset $D_{w}=D_{l}\cup {\left\{\left(x_{j},y_{j}\right)\right\}}_{j=n+1}^{n+m}$ is constructed. The loss function is redefined:

(12) $$L_{new}\left(\theta \right)=\frac{1}{n+m}\sum_{i\in D_{new}}\left({\left(y_{i}-f_{\theta }\left(x_{i}\right)\right)}^{2}\right).$$

If i≤n, $y_{i}$ is the true value of the labeled data; if i>n, $y_{i}$ is the pseudo-label $y_{j}$. The parameter θ is updated using an optimization algorithm. The gradient of $L_{new}\left(\theta \right)$ is found with respect to θ. Let $z_{i}={\left(y_{i}-f_{\theta }\left(x_{i}\right)\right)}^{2}$.

(13) $$\frac{\partial L_{new}\left(\theta \right)}{\partial \theta }=\frac{1}{n+m}\sum_{i\in D_{new}}\cdot \frac{\partial z_{i}}{\partial \theta }.$$

When $z_{i}={\left(y_{i}-f_{\theta }\left(x_{i}\right)\right)}^{2}>0$,

(14) $$\frac{\partial L_{new}\left(\theta \right)}{\partial \theta }=\frac{2}{n+m}\sum_{i\in D_{new}}\left(y_{i}-f_{\theta }\left(x_{i}\right)\right)\cdot \frac{\partial f_{\theta }\left(x_{i}\right)}{\partial \theta }.$$

The model update rule is $\theta =\theta -\beta \nabla_{\theta }L_{new}\left(\theta \right)$, where β is the new learning rate. This process can be iterated repeatedly: the model and pseudo-labels are continuously optimized to improve the performance of the model in semi-supervised learning.

#### 3.2.2. Multi-Fidelity Data Fusion Model Based on Pseudo-Label Semi-Supervised Learning

**Figure 3:**
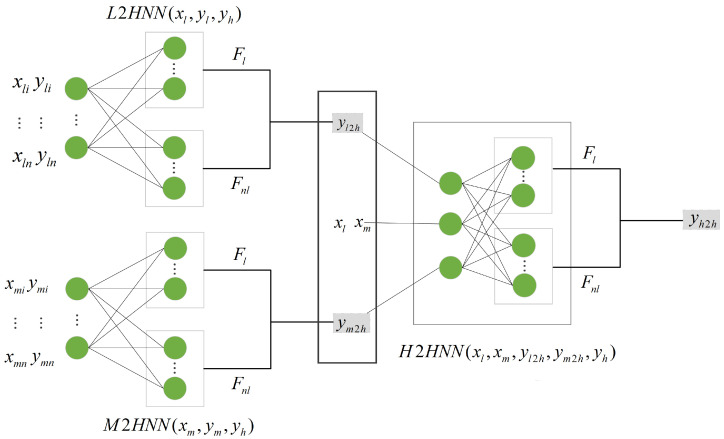
SSLMF schematic diagram. $L2HNN(x_{l},y_{l},y_{h})$ and $M2HNN(x_{m},y_{m},y_{h})$ represent the network modules that map low-fidelity and medium-fidelity data to high-fidelity data, respectively. $H2HNN(x_{l},x_{m},y_{l2h},y_{m2h},y_{h})$ denotes the high-to-high mapping. $F_{l}$ denotes the linear term, while $F_{nl}$ represents the nonlinear term. Additionally, $y_{l2h}$, $y_{m2h}$, and $y_{h2h}$ denote the fusion results from low-to-high-fidelity, medium-to-high-fidelity, and the final high-to-high-fidelity fusion, respectively.

The framework of the SSLMF model based on pseudo-label semi-supervised learning is now described. The model comprises three network modules, each containing two fully connected neural networks. One neural network uses an activation function, while the other does not. The framework is shown in Figure 3. Two fully connected neural networks in the $L2HNN(x_{l},y_{l},y_{h})$ network module are used to determine the linear and nonlinear relationships between low- and high-fidelity data and to perform low-to-high-fidelity data fusion and obtain the low to high fidelity representation $y_{l2h}$ after output fusion. Similarly, two fully connected neural networks in the $M2HNN(x_{m},y_{m},y_{h})$ network module search for the correlations between medium- and high-fidelity data and perform medium-to-high-fidelity data fusion, generating medium to high fidelity representations $y_{m2h}$. Finally, the $H2HNN(x_{l},x_{m},y_{l2h},y_{m2h},y_{h})$ network module fuses the first two networks to obtain the fusion result of $y_{l2h},y_{m2h}$, i.e., medium- and low-fidelity data, with $x_{l},x_{m}$ as input, and identifies linear and nonlinear relationships in $y_{l2h},y_{m2h}$. Each network module weights the linear and nonlinear relationships between the data through the weights ρ obtained by training. The model learns the information between different data types through three network modules, and obtains fusion results containing low-, medium-, and high-fidelity information.

Each network model is trained by a semi-supervised learning method that generates pseudo-labels. In the training process, the existing multi-fidelity data are divided into datasets by the following methods: (1) medium- and low-fidelity data corresponding to high-fidelity data are used as labeled data; (2) other medium- and low-fidelity data (i.e., without corresponding high-fidelity data) are used as unlabeled data. The dataset is divided through this method, and the semi-supervised learning method of generating pseudo-labels is used to train the model. Thus, a fusion model with good performance can be developed from very few high-fidelity labeled data. The problem of imbalanced data volumes is solved, and the unlabeled data are fully utilized to achieve better model performance. The training process of each network module in the constructed model is shown in Figure 2.

In SSLMF, the network modules $L2HNN(x_{l},y_{l},y_{h})$ and $M2HNN(x_{m},y_{m},y_{h})$ map low- and medium-fidelity data to high-fidelity data, respectively, and construct the correlations between low-, medium-, and high-fidelity data as follows:

(15) $$F_{l2h}\left(x_{l},y_{l}\right)\to y_{h},$$

(16) $$F_{m2h}\left(x_{m},y_{m}\right)\to y_{h},$$

where $x_{l},y_{l}$ represents low-fidelity data, $x_{m},y_{m}$ represents medium-fidelity data, and $y_{h}$ represents high-fidelity data. $F_{l2h}\left(\cdot \right)$ and $F_{m2h}\left(\cdot \right)$ are the functions relating medium- and low-fidelity data to high-fidelity data, respectively, which are obtained by training network modules $L2HNN(x_{l},y_{l},y_{h})$ and $M2HNN(x_{m},y_{m},y_{h})$. The mapping functions are expressed as follows:

(17) $$Q\left(F_{l2h}\left(x_{l},y_{l}\right),y_{h}\right)=y_{l2h},$$

(18) $$P\left(F_{m2h}\left(x_{m},y_{m}\right),y_{h}\right)=y_{m2h},$$

where $y_{l2h},y_{m2h}$ are the low-to-high and medium-to-high data fusion results, respectively, Q(·) is the mapping function from low-fidelity data to high-fidelity data, and P(·) is the mapping function from medium-fidelity data to high-fidelity data. The network module $H2HNN(x_{l},x_{m},y_{l2h},$$y_{m2h},y_{h})$ constructs the correlation between $y_{l2h}$, $y_{m2h}$, and $y_{h}$ as follows:

(19) $$F_{h2h}\left(x_{l},x_{m},y_{l2h},y_{m2h}\right)\to y_{h},$$

where $F_{h2h}\left(\cdot \right)$ represents the relationship between the $y_{l2h}$ fusion result, containing low- and high-fidelity data, and the $y_{m2h}$ fusion result, containing medium- and high-fidelity data. The mapping relationship between Q(·) and P(·) can be expressed as follows:

(20) $$K\left(Q\left(\cdot \right),P\left(\cdot \right),y_{h}\right)=y_{h2h},$$

where $y_{h2h}$ represents the fusion result of $y_{l2h},y_{m2h}$ and the high-fidelity data $y_{h}$, and K(·) represents the mapping function between Q(·), P(·), and the high-fidelity data. The mapping function K(·) contains information on the low-, medium-, and high-fidelity data, which enables the proposed model to obtain more accurate fusion results.

To prevent pseudo-labels from dominating the training set and introducing excessive noise, we impose a stopping criterion based on data volume. Specifically, the total number of pseudo-labeled samples is constrained to not exceed the combined number of real low-fidelity and high-fidelity samples. This strategy helps maintain training stability and avoids error amplification during the iterative pseudo-label generation process.

In all tasks, the labeled data consists of a limited number of high-fidelity samples that are originally available with ground truth annotations. These labeled samples are used to pre-train a model, which is then applied to unlabeled low-fidelity data to generate pseudo-labels. These pseudo-labeled samples are subsequently included in training via a semi-supervised loss. This partitioning strategy reflects practical conditions where high-fidelity labeled data are expensive to acquire, while unlabeled multi-fidelity data are relatively abundant.

The pseudo-labels used in our method are generated by a model that has been pre-trained on a limited amount of high-fidelity data. Although these labels may not be perfectly accurate, they inherently encode the underlying trends and structural patterns of the high-fidelity source. Rather than relying solely on precision, the multi-fidelity fusion approach focuses on enriching the model with diverse information sources, thereby improving feature representation and robustness. This design helps mitigate the risk of error propagation typically associated with pseudo-labels, as the model benefits from consistent pattern signals across fidelity levels.

## 4. Example Verification and Results Analysis

The method was initially validated on nonlinear continuous benchmark functions and subsequently evaluated for its performance in image super-resolution reconstruction. Experimental results demonstrate that the method consistently achieves excellent outcomes in such tasks.

To ensure stable training behavior within the pseudo-labeling framework, we adopt several practical constraints. First, pseudo-labels are generated using a pretrained model on limited high-fidelity data, which helps preserve essential structural patterns. While these labels may contain some noise, they offer valuable guidance for downstream learning. To prevent error propagation, we constrain the number of pseudo-labeled samples such that it does not exceed the total number of real high- and low-fidelity samples. This balance ensures that the model is not overwhelmed by synthetic labels. In practice, we observed that the training process remains stable across runs, and the pseudo-labeling loop does not introduce noticeable instability.

### 4.1. Function Approximation

Following the function examples using dual-fidelity data as a reference, this paper presents examples in which low-, medium-, and high-fidelity data are fused. The results verify the effectiveness of the proposed multi-fidelity modeling method in approximating continuous functions based on nonlinear correlations.

The performance of the proposed method is compared with the MFDNN [40] model, and ablation experiments are conducted on the benchmark function examples to further demonstrate the effectiveness of semi-supervised learning.

The model performance is evaluated using the relative error:

(21) $$R=\frac{∥y_{Model}^{i}-y_{Exp}^{i}{∥}_{F}^{2}}{∥y_{Exp}{∥}_{F}^{2}},$$

where the relative error *R* is an indicator of the relative size of the error between the predicted and true values. The smaller the value of *R*, the better the model’s predictive performance.

In our SSLMF framework, the weight parameter ρ controls the contribution of linear and nonlinear terms in the fidelity fusion module. We treat ρ as a learnable scalar parameter, initialized to 0.5, and include it in the trainable parameter set passed to the optimizer. During training, ρ is updated via standard backpropagation based on the reconstruction loss, allowing the model to adaptively learn the optimal balance between linear and nonlinear components.

All experiments are implemented in PyTorch 2.0.1 and trained on a single NVIDIA RTX 4070Ti GPU (NVIDIA Corporation; Santa Clara, CA, USA). We used the Adam optimizer with an initial learning rate of 1 × $10^{-4}$, $\beta_{1}=0.9$, and $\beta_{2}=0.999$. All input images were normalized to [0,1] and randomly cropped during training to size 128×128. For all models we adopt a fully convolutional structure with five convolutional layers in both the encoder and decoder. The number of channels for each layer is [64,128,256,128,64]. All convolution layers use 3×3 kernels with stride 1 and padding 1, followed by ReLU activations. For image reconstruction tasks, the input is a 64×64 low-fidelity image, and the output is a predicted high-fidelity image of the same resolution. Batch normalization is applied after each layer to stabilize training.

#### Nonlinearly Related Continuous Functions

This section considers a continuous function with a nonlinear correlation [49] to prove that the proposed model can capture complex nonlinear relationships. The multi-fidelity data are given by the following functions:

(22) $$\mu_{l}\left(x\right)=\sin\left(8\pi x\right),x\in \left[0,1\right],$$

(23) $$\mu_{m}\left(x\right)=\left(x-\sqrt{2}\right)\mu_{l}\left(x\right),$$

(24) $$\mu_{h}\left(x\right)=\left(x-\sqrt{2}\right){\mu_{l}}^{2}\left(x\right).$$

For this example, 10 medium- and low-fidelity data points and 10 high-fidelity data points are randomly selected, and the sampling points are not evenly distributed. Table 1 summarizes the division of data.

**Figure 4:**
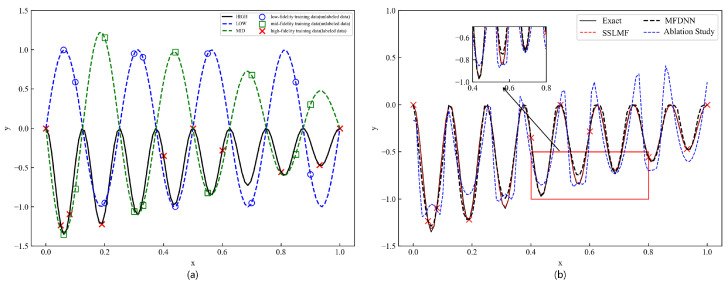
An approximation of a continuous function with nonlinearly correlated multi-fidelity data. In (**a**), the green, black, and blue curves represent the medium-fidelity, low-fidelity, and high-fidelity data, respectively. The green squares and blue hollow circles denote the unlabeled medium-fidelity and low-fidelity sampling points, while the red crosses represent the high-fidelity data points. In (**b**), the red dashed line indicates the predictions of the proposed model, the blue dashed line represents the results obtained using only the labeled data, and the black solid line denotes the ground truth. The black dashed line represents the predictions of the MFDNN method.

The locations of the sampling points are shown in Figure 4a. The linear part of the network uses two hidden layers, each with 10 neurons, while the nonlinear part uses five hidden layers, each containing 60 neurons. The settings of the three network modules are consistent. The weight attenuation is set to $1\times 10^{-5}$ and the learning rate is $1\times 10^{-4}$. The experimental results are shown in Figure 4.

To evaluate the effect of labeled high-fidelity training data quantity on model performance, we progressively reduce the proportion of available high-fidelity labeled samples used for training. For each reduced-data setting, the model is retrained from scratch using only the selected subset of labeled samples. After training, the model’s reconstruction error is measured on a fixed, independent test set to ensure consistent evaluation. This procedure allows us to systematically assess how decreasing labeled data impacts the reconstruction accuracy.

**Figure 5:**
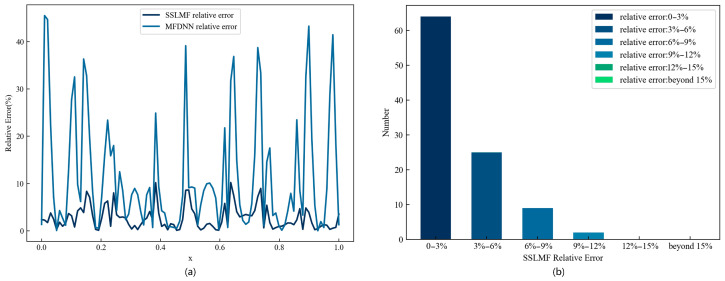
A comparative analysis of relative errors. (**a**) A prediction relative error curve. (**b**) A relative error statistical histogram.

As shown by the red dotted line in Figure 4b, even when the trend of the medium- and low-fidelity data is opposite to that of the high-fidelity data, the network still produces accurate predictions, whereas the results obtained using only labeled data do not fit the real curve well. Furthermore, within the region of 0.4<x<0.8, demarcated by the red box in Figure 4b, no high-fidelity data were utilized in this study. Despite this absence, the results clearly demonstrate that the proposed method outperforms the MFDNN in terms of accuracy. This superior performance can be attributed to the method’s more comprehensive utilization of the valuable information contained within unlabeled data. By effectively leveraging this information during the training process, the proposed method can maintain excellent performance even in the absence of high-fidelity data, highlighting its robustness and efficiency in data-driven modeling. Next, a visual comparison chart of relative errors between the proposed method and the comparative methods was obtained. A systematic statistical analysis of the relative errors of the proposed method was conducted to more intuitively represent the differences in errors among different methods, as shown in Figure 5.

We can see from Figure 5a that selecting only a small number of high-fidelity data points increases the relative error significantly in the range of 0.1<x<0.3 and 0.4<x<0.8. This is because no high-fidelity data points are selected in this range. The maximum relative error reaches approximately 10%. Figure 5b shows that the overall relative error is concentrated in the range 0–6%, indicating that the model accurately captures the complex nonlinear correlations between the data. It has significant advantages over MFDNN and demonstrates excellent performance.

The results for a function with a nonlinear correlation demonstrate that SSLMF maintains excellent fitting ability when there are very few high-fidelity data points in the training dataset. This method uses low-, medium-, and high-fidelity data to construct the model, with the multiple fidelities cooperating closely and complementing each other. The overall result is a closed loop that makes up for the lack of information and improves the prediction accuracy of the model. Using semi-supervised learning to generate pseudo-labels allows accurate prediction results to be obtained from very few high-fidelity data points during the training process. The proposed SSLMF model can learn useful information from unlabeled medium- and low-fidelity data, providing the distribution trends and characteristics. This additional information helps reduce the risk of model overfitting. By using unlabeled data for training, more comprehensive data distribution information can be extracted to improve the robustness and accuracy of the model.

**Figure 6:**
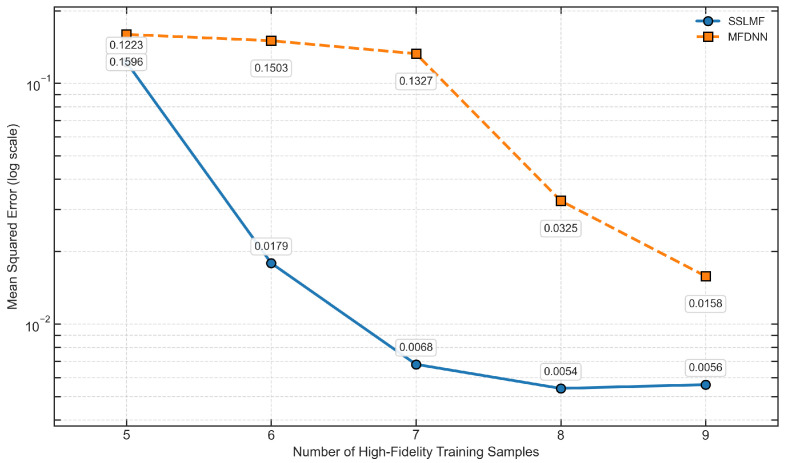
Comparison of error metrics with varying amounts of high-fidelity training data.

As shown in Table 2 and Figure 6, the reconstruction error of all methods increases as the number of labeled high-fidelity training samples decreases. This is expected, as reduced supervision typically leads to diminished modeling accuracy. However, the proposed SSLMF method demonstrates a significantly slower error growth rate compared to MFDNN, indicating stronger resilience to labeled data scarcity. This highlights the benefit of integrating semi-supervised learning into the multi-fidelity modeling process. The pseudo-labels generated by the pretrained model capture meaningful data structures and feature patterns from both high- and low-fidelity sources. These pseudo-labels enrich the training signal and enable the final model to achieve more accurate predictions on the test set, even with limited labeled data.

### 4.2. High-Resolution Image Reconstruction

Having verified that the proposed model achieves good accuracy in approximating nonlinearly related continuous functions, its performance in image super-resolution reconstruction tasks is now evaluated. To comprehensively assess the quality of the reconstructed images, two commonly used objective evaluation metrics are adopted: the peak signal-to-noise ratio (PSNR) and the structural similarity index measure (SSIM).

The PSNR measures the pixel-level error between the reconstructed and reference images; a higher value indicates better reconstruction quality. It is usually expressed in decibels (dB) and is a widely used performance evaluation metric in image processing. The SSIM, on the other hand, simulates the human visual system’s perception of structural changes in images. It takes into account luminance, contrast, and structural features. The SSIM value ranges from 0 to 1, with values closer to 1 indicating higher structural similarity between images.

In addition, to more intuitively analyze the distribution of local errors, this section presents absolute error maps and error distribution profiles. The absolute error map visually displays the magnitude of reconstruction errors at each pixel using color coding, while the error distribution profile shows the frequency of different pixel error magnitudes. The vertical axis of the profile is plotted on a logarithmic scale to more clearly reflect the frequency characteristics across different error ranges.

It is worth noting that under the current working conditions, the training parameters are set as follows: the learning rate is 0.0001 and the number of iterations is 10,000. Each sub-model is composed of a fully connected neural network, containing a total of 550,000 trainable parameters, and the entire training process is also deployed on the NVIDIA GeForce RTX 4070Ti GPU. The total training time is approximately 2 h.

#### 4.2.1. Data Construction

**Figure 7:**
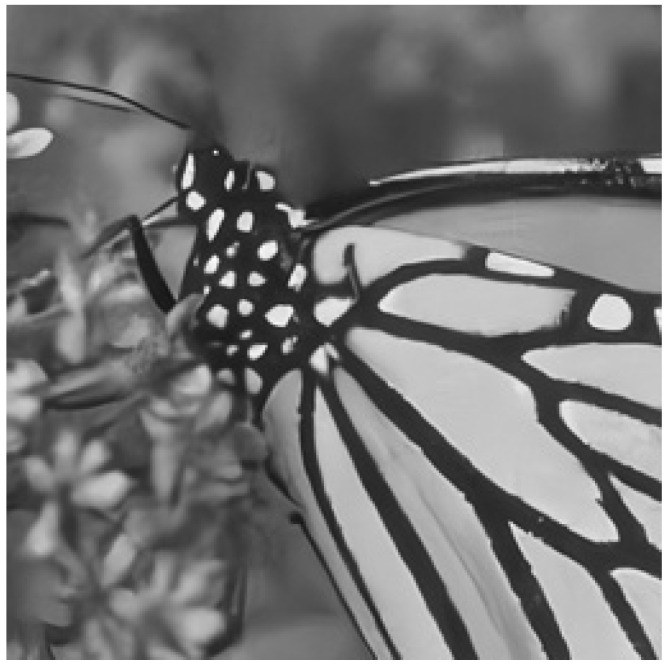
Original high-definition image.

This section uses a high-definition grayscale image as the dataset for an image super-resolution reconstruction study. The original image is shown below in Figure 7.

**Figure 8:**
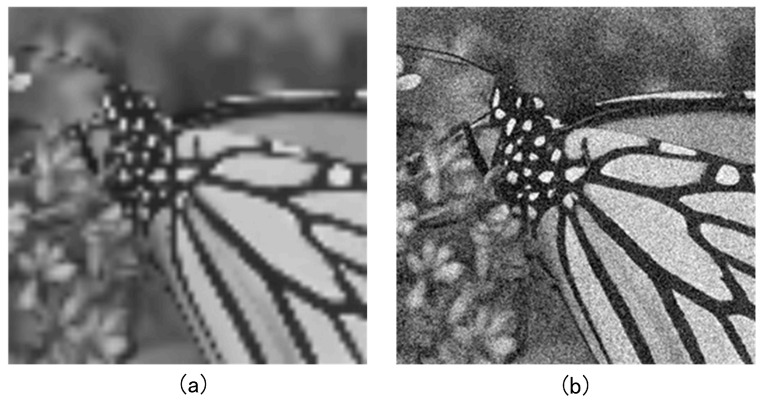
Low- and medium-fidelity resolution images, where (**a**) is the low-fidelity resolution image and (**b**) is the medium-fidelity resolution image with added noise.

To validate the effectiveness of the proposed method in image super-resolution reconstruction, the original high-resolution image is downsampled to reduce its resolution, and the resulting low-resolution image is used as low-fidelity data. Then, Gaussian noise is added to the original image, and the result is used as medium-fidelity data. The obtained data is shown below in Figure 8. For the data, a random sampling strategy is used to split the available data into labeled and unlabeled subsets. To ensure reproducibility, we fix the random seed. As the tasks are regression-oriented rather than classification-based, stratified sampling is not required.

**Figure 9:**
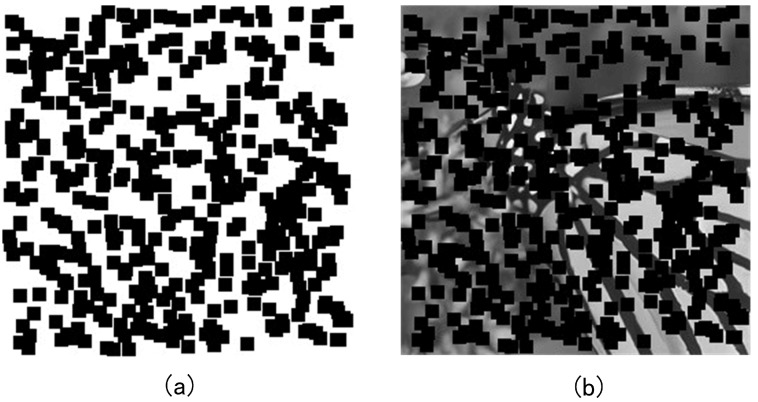
High-fidelity resolution image data partitioning, where (**a**) is the selected 80% mask, and (**b**) is the original image after masking, which is used as the high-fidelity resolution image.

Additionally, random sampling is used to select 80% of the mask on the original high-resolution image to block parts of the original image. The unmasked portion is used as high-fidelity data, as shown below in Figure 9.

The medium- and low-fidelity resolution images corresponding to the unmasked high-fidelity resolution image in Figure 9 are used as labeled data, while the remaining medium- and low-fidelity resolution images corresponding to the masked areas are used as unlabeled data. This results in a training set with a labeled-to-unlabeled data ratio of 1:4.

#### 4.2.2. Super-Resolution Experimental Analysis

**Figure 10:**
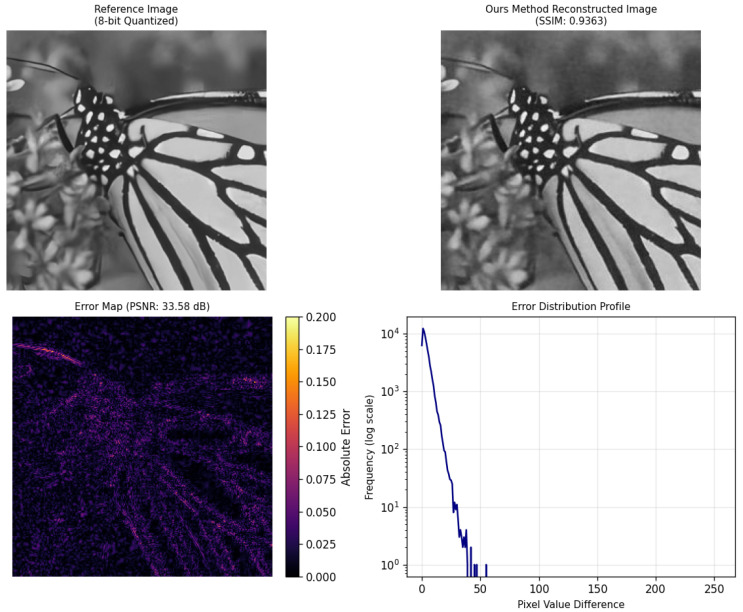
The experimental results and evaluation metrics of the super-resolution reconstruction using the proposed method.

**Figure 11:**
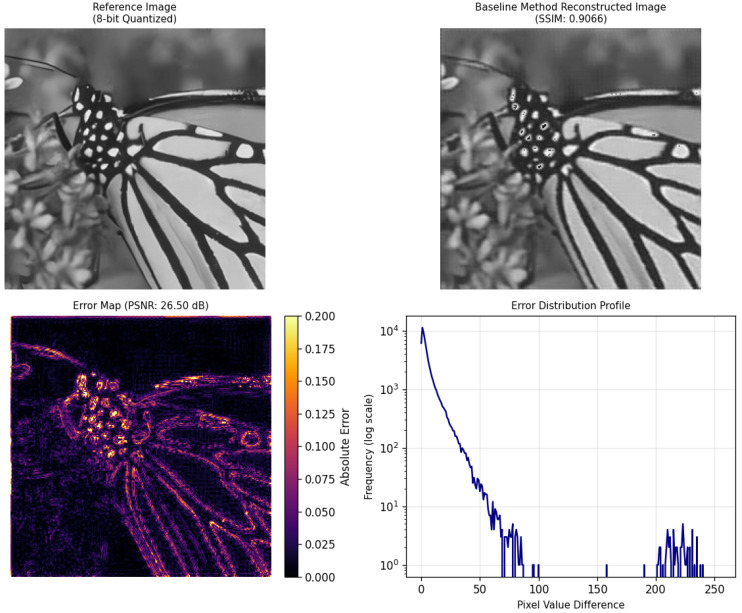
Experimental results and evaluation metrics of super-resolution reconstruction using the benchmark method.

**Figure 12:**
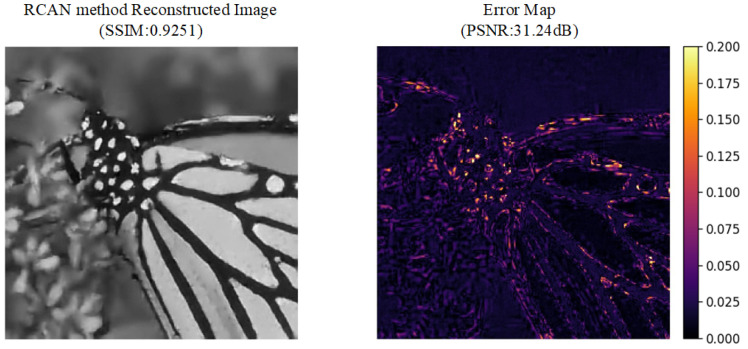
Experimental results and evaluation metrics of super-resolution reconstruction using the RCAN method.

This section uses the dataset partitioned using the aforementioned method for model training. Although a large amount of training data is selected, the labeled data is limited, and a significant amount of unlabeled data is used for model training to validate the effectiveness of the proposed method. Finally, the low-fidelity resolution image shown in Figure 8a is reconstructed to obtain the original high-resolution image shown in Figure 7. The experiment uses a semi-supervised learning approach to construct the model with the built data, and the results are compared with existing methods. The experimental results are shown in Figure 10, Figure 11 and Figure 12. In this experiment, a semi-supervised learning approach was adopted to construct a model using the built dataset, and the results were systematically compared with the existing MFDNN method and RCAN method [50]. To adapt to the input requirements of the MFDNN method, images were processed into one-dimensional vectors for multi-fidelity super-resolution reconstruction. Although this data transformation was performed, the fundamental nature of the task remains multi-fidelity data fusion, which aims to exploit the complementary information across different fidelity levels to enhance reconstruction performance.

Figure 10, Figure 11 and Figure 12, respectively, show the reconstruction results and corresponding error analyses of the proposed method and the benchmark method on the same reference image (8-bit quantization). In terms of evaluation metrics, the proposed method outperforms the benchmark method and RCAN method in both PSNR and SSIM, with the specific results shown in Table 3 below.

Compared with the benchmark method, the proposed method improves the PSNR by 7.08 dB and the SSIM by 0.0297, indicating a significant advantage in noise suppression and structural preservation. The benchmark method shows a high SSIM but low PSNR, suggesting the presence of Gaussian noise contamination. Compared with the RCAN method, the proposed method still maintains a higher PSNR and SSIM. The proposed method achieves a PSNR of 33.58 dB and an SSIM of 0.9363, demonstrating that the reconstructed image reaches a high-quality, high-resolution level.

From the perspective of subjective visual quality, the reconstructed image produced by the proposed method appears more natural in terms of texture details, edge structures, and background transitions. It effectively preserves the clear textures of the butterfly’s wings as well as the details of the background vegetation. In contrast, the images reconstructed by the benchmark method and RCAN method exhibit noticeable blocking artifacts and blurred details, with severe distortions in high-frequency regions such as the wing patterns. The error map further validates these qualitative observations: The errors of the proposed method are mainly concentrated in local edge areas, with relatively low overall error magnitudes. On the other hand, the benchmark method shows large high-error regions across the image, which appear as bright error patches. For the RCAN method, although it has enhanced its detail recovery ability through channel attention and deep residual groups, it can still be seen from the error map that it is insufficient in handling complex backgrounds or non-uniform textures. This might be due to the channel attention being overly dependent on certain feature channels, resulting in incorrect magnification in local areas.

Moreover, the error distribution profile reveals that the proposed method’s errors are primarily concentrated in the low-amplitude range (pixel differences less than 30), with frequencies decreasing rapidly as error magnitude increases. This is indicative of desirable reconstruction characteristics. In contrast, the benchmark method and RCAN method exhibit a greater number of medium- to high-amplitude errors and still show significant occurrences in the high-amplitude range (pixel differences greater than 100), indicating poorer reconstruction stability. In comparison, the proposed method delivers superior performance.

The benchmark method introduces a significant amount of Gaussian noise during super-resolution reconstruction, primarily because it fails to properly handle and utilize the information provided by the low- and medium-fidelity data during fusion, resulting in information loss. In contrast, the proposed method effectively leverages low- and medium-fidelity data through semi-supervised learning, enabling it to maintain excellent performance even in scenarios with sharp data variations. Meanwhile, the prediction time of our method on an NVIDIA GeForce RTX 4070Ti is only 0.013 s.

In addition, we conducted a systematic analysis of the sensitivity of different models to the learning rate, and the results are shown in Table 4. The experimental results show that our model exhibits strong robustness to changes in the learning rate. Specifically, when the learning rate varies within the range of [1 × $10^{-4}$, 1 × $10^{-2}$], the variance of the PSNR index is only 5.71 dB, significantly lower than 5.90 dB of the benchmark method and 6.24 dB for RCAN. This result fully demonstrates that our method has better adaptability and stability in the selection of learning rates, and can effectively reduce performance fluctuations caused by improper setting of learning rates.

Overall, the experimental results demonstrate that the proposed multi-fidelity data fusion method based on pseudo-label semi-supervised learning can achieve satisfactory prediction results using only a small amount of labeled data, with the aid of a large volume of unlabeled data. This method exhibits strong performance when dealing with complex aerodynamic heating data and proves especially effective in high-resolution image reconstruction tasks. The model construction and validation show that the proposed approach not only overcomes the limitations of existing data fusion models under limited high-fidelity data but also demonstrates strong adaptability and accuracy in practical engineering applications such as image super-resolution reconstruction.

In addition, through the semi-supervised learning strategy, the proposed method fully exploits the information provided by the low- and medium-fidelity data, effectively addressing the limitations caused by the scarcity of high-fidelity data. This significantly enhances the model’s robustness and prediction accuracy. Even under sharp data variations and outliers, the proposed method maintains excellent predictive performance, ensuring broad applicability to complex nonlinear problems. It shows promising application potential and practical significance in high-resolution reconstruction tasks.

### 4.3. Audio Spectrogram Reconstruction

Image reconstruction methods not only play a crucial role in traditional computer vision tasks but also demonstrate broad application potential across various interdisciplinary domains. For instance, in audio-related tasks, researchers often convert audio signals into spectrograms, treating them as images for processing. This enables functionalities such as audio restoration, enhancement, or separation. Representing audio in spectrogram form allows reconstruction algorithms originally developed for image processing to be naturally transferred and applied to modeling and recovering audio signals.

Building on this idea, the semi-supervised image reconstruction method proposed in this work exhibits strong crossmodal generalization potential. It can achieve effective modeling even with limited high-fidelity labeled data. To further validate the adaptability and robustness of this method in complex cross-domain tasks, this subsection presents a case study on audio restoration. By reconstructing spectrograms, we systematically evaluate the method’s performance in the audio domain, thereby demonstrating its interdisciplinary applicability across heterogeneous data types such as images and audio.

#### 4.3.1. Case Study Description

Specifically, this study constructs an audio restoration scenario with practical relevance to validate the proposed method’s crossmodal reconstruction capability under weak supervision. In the experiment, three types of degraded audio are used to build the model: (1) audio mixed with mobile phone ringtone interference, (2) audio overlaid with exhibition hall ambient noise, (3) audio containing noticeable stuttering artifacts. The objective of the experiment is to recover clean, continuous, and original audio signals from these three types of degraded inputs. Notably, the high-quality reference audio used for evaluation is not involved in the model training process, significantly increasing the task difficulty and more closely reflecting real-world weakly supervised restoration scenarios.

The proposed image reconstruction method is adopted to address the above restoration task, and design redundant convolutional encoder (R-CED) [51] methods for comparison. First, all three types of audio signals are transformed into spectrograms, which express the audio features under different degradation conditions in image form. This enables cross-modal feature alignment and unified modeling. Next, under the proposed semi-supervised image reconstruction framework, a multi-fidelity data fusion strategy is integrated to collaboratively model and reconstruct the spectrograms of the three audio types. This approach effectively leverages the complementary information across different audio samples, enabling accurate approximation and recovery of the original spectrogram representation.

To further verify the robustness of our method in the audio reconstruction task, we conducted an additional set of independent repeated experiments. Specifically, we fixed the data partitioning (training, validation, and test sets) and repeated the entire training and evaluation process 10 times, each with a different random initialization. The variance across the 10 runs is reported to assess the stability and consistency of the proposed method. The low variance observed in the performance metrics confirms that the model exhibits strong robustness under random initialization conditions.

**Figure 13:**
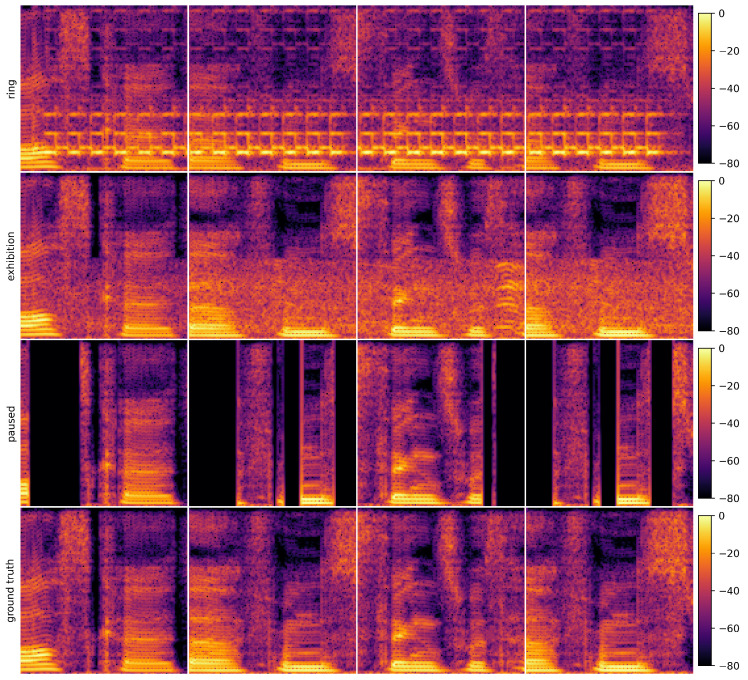
Spectrograms of three degraded audio types and clean reference audio at four random time frames.

Figure 13 presents four groups of spectrogram samples, corresponding to four randomly selected time frames, to intuitively compare the spectral differences between the original audio and the three degraded versions. The four vertical columns correspond to four random time frames. The first row shows the spectrograms with added ringtone interference, where sudden energy spikes are observed in the mid-to-high-frequency range. These non-stationary and abrupt disturbances simulate sudden ringtone noise in daily life. The sudden bright yellow patches in the figure correspond to the high-frequency characteristic frequency band of the ringtone. The second row displays spectrograms with exhibition hall background noise, characterized by widespread low- to mid-frequency components that are continuous and persistent, representing typical open-space ambient noise. The continuous orange-red areas in the image reflect the principal components of environmental noise. The third row shows spectrograms with stuttering, where various degrees of silent regions appear across time frames. This reflects interruptions or frame drops in the audio, simulating issues such as unstable network transmission or buffer loss. The deep black stripes in the image mark the time intervals of audio interruptions. The fourth row provides the spectrograms of clean reference audio, featuring smooth, continuous, and complete time-frequency structures, serving as the ideal benchmark.

This case study demonstrates the robustness and generalization ability of the proposed method in handling different types of degraded audio. It also substantiates the interdisciplinary potential of image reconstruction algorithms when applied to audio spectrogram reconstruction tasks.

#### 4.3.2. Modeling and Results

In the context of audio restoration, we focus on a representative degradation scenario: stuttering audio. Such audio exhibits silent segments within the temporal sequence, representing interruptions or missing data, while the non-silent segments maintain high consistency with the clean reference audio. Leveraging this structural characteristic, we treat the non-silent portions of the stuttering audio as reliable sources of supervision and construct them as soft labels to guide the model’s semi-supervised learning process. This strategy effectively addresses the challenge of limited access to full, high-quality audio samples in the training set, enabling viable modeling under complex degradation conditions.

Specifically, we first generate a binary spectrogram mask based on the spectral characteristics of the stuttering audio, where non-silent regions are marked as 1 and silent regions as 0. Based on this mask, the non-silent regions are used for supervised training, while the silent regions are designated for semi-supervised modeling. In the supervised learning phase, the non-silent regions of the stuttering audio are paired with spectrograms from the other two types of degraded audio (those containing ringtone interference and exhibition background noise) at corresponding time positions to form input–output training pairs. The spectrograms of the degraded audio serve as inputs, while the high-quality spectrogram segments from the stuttering audio act as output labels, creating partially supervised data. This allows the model to learn the mapping between various degradation types and the corresponding clean spectrogram structures.

On this basis, for the silent regions in the stuttering audio where no ground-truth labels are available, we employ a pseudo-label generation and iterative refinement mechanism to facilitate semi-supervised learning. During training, the model uses its current mapping function to generate preliminary predictions in the silent regions, which are treated as pseudo-labels. These pseudo-labels are iteratively refined through continuous training, allowing the model to gradually approximate the true spectrogram structure in these regions. This self-training process equips the model to estimate plausible content in previously unseen regions, effectively unifying the goals of multi-source low-fidelity data fusion and high-fidelity reconstruction. Consequently, the model’s generalization and reconstruction performance in diverse degradation scenarios are significantly enhanced.

**Figure 14:**
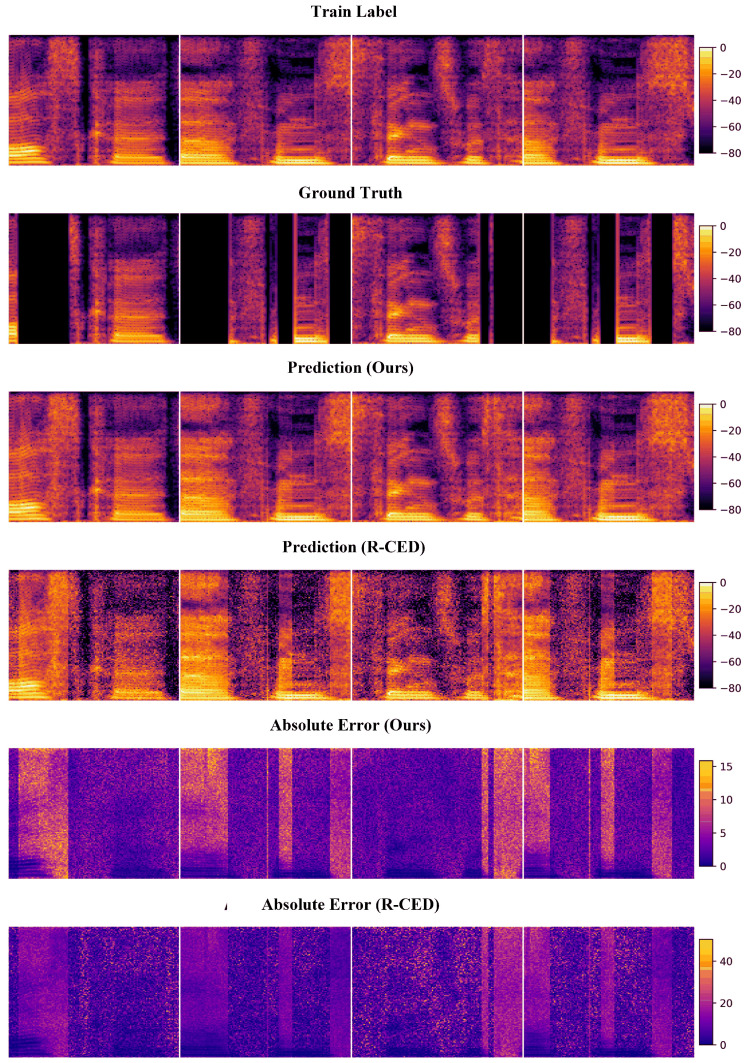
Modeling results at four random time frames.

The reconstruction results of the proposed SSLMF method and the R-CED model are visually compared in Figure 14, respectively. These figures illustrate the predicted outputs for representative audio samples. Table 5 presents the evaluation outcomes of PSNR and SSIM. Four randomly selected time frames are shown, each illustrating the spectrogram of the original stuttering audio, the model’s prediction, and the clean reference audio. As seen, the stuttering spectrograms contain distinct silent regions. The model’s predictions closely match the ground-truth spectrogram in the supervised (non-silent) areas, demonstrating strong learning performance in regions with label support. In the silent regions, while discrepancies from the ground-truth still exist, the predicted spectrograms maintain high continuity and contextual consistency. There are no evident structural disruptions or noisy artifacts, indicating that the pseudo-label refinement strategy plays a constructive role in reconstructing missing information. It is clearly observable that SSLMF produces reconstructions with fewer artifacts, sharper details, and higher structural fidelity than R-CED, especially in complex or high-frequency regions. To support these visual observations, Table 5 provides a quantitative comparison of the reconstruction quality using metrics such as PSNR and SSIM. Across all evaluated samples, SSLMF consistently achieves higher scores, confirming its superior performance over R-CED. The results demonstrate that our method not only enhances reconstruction accuracy but also improves perceptual quality, which is critical in real-world multi-fidelity tasks.

**Figure 15:**
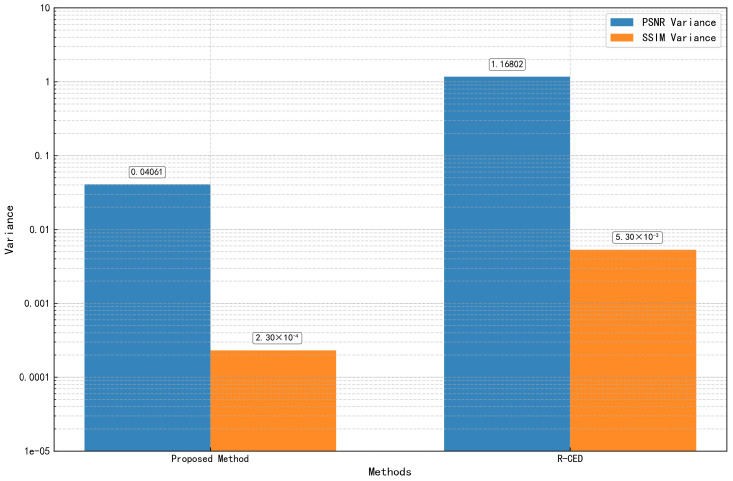
Comparison of PSNR and SSIM variances across different methods in audio task.

To further assess the statistical robustness of the proposed method, we conducted 10 independent training and evaluation runs on the audio reconstruction task, under fixed data partitioning. The quantitative results of these repeated experiments are summarized in Table 6, while the corresponding variance trends are visualized in Figure 15. From the statistical results, the proposed SSLMF method demonstrates significantly higher stability compared to R-CED. Specifically, the variance of PSNR across 10 runs for SSLMF is 0.04061, and the variance of SSIM is 2.30 × $10^{-4}$. In contrast, the commonly used R-CED exhibits a PSNR variance of 1.16802 and an SSIM variance of 5.3 × $10^{-3}$, indicating substantially less consistency across runs. This improvement in robustness can be attributed to the semi-supervised pseudo-labeling mechanism embedded in SSLMF, which introduces diverse and informative data features during training. The enriched feature representations enhance the model’s generalization ability and help stabilize the learning process, thereby leading to more consistent and accurate reconstructions across different runs.

The experimental results confirm that the proposed method exhibits strong learning and reconstruction capabilities even under real-world scenarios with incomplete data and sparse labels. The method not only leverages the limited high-quality label data available in stuttering audio but also integrates information from multiple degradation sources for effective modeling. Moreover, it shows robust predictive performance in unsupervised regions, reflecting strong crossmodal transferability and resilience in restoration. This audio spectrogram reconstruction task provides empirical evidence of the method’s cross-domain applicability. It demonstrates its feasibility in extending from image reconstruction to audio processing and lays a methodological and experimental foundation for its application in broader multimodal fusion and complex degradation challenges.

## 5. Conclusions

This study has validated the effectiveness of the multi-fidelity data fusion method based on pseudo-label semi-supervised learning (SSLMF) in data-scarce scenarios. This innovative approach leverages a large amount of unlabeled data to achieve satisfactory prediction results using only a small amount of labeled high-fidelity samples, effectively addressing the bottleneck of insufficient high-quality data in practical applications. The method demonstrates outstanding performance in handling complex multi-fidelity high-resolution image reconstruction tasks, effectively addressing information loss and irreversibility issues during the transition from low to high resolution. It exhibits strong adaptability and accuracy, achieving promising results in practical engineering applications.

By integrating MFDF with SSL, SSLMF uniquely combines global structural constraints from low-fidelity data and pseudo-label guidance from unlabeled data. This synergy not only compensates for the scarcity of high-fidelity data but also enables the model to capture both linear and nonlinear relationships across data fidelities, significantly enhancing robustness in data-scarce scenarios. The synergy among low-, medium-, and high-fidelity data allows different types of data to complement each other, forming a closed-loop system that mitigates information loss. This closed-loop approach proves particularly effective in addressing challenges such as rapid data variation and the presence of outliers, maintaining excellent predictive performance even in complex environments. Notably, the pseudo-label mechanism iteratively refines unlabeled low/medium-fidelity data, transforming it into effective supervision signals—this is the key to overcom data imbalance in scenarios where high-fidelity samples are scarce.

In our current experimental design, more attention is paid to controlled scenarios, and datasets that are artificially degraded or masked are used. The original intention of setting this up was to more clearly verify the core mechanism and basic performance of semi-supervised learning in multi-fidelity data fusion methods in a relatively pure environment, eliminate the influence of complex interfering factors on the effectiveness of the method itself, and lay a reliable foundation for subsequent practical application research. However, data in real-world scenarios often contains complex noise, various forms of distortion, and uncontrollable interference factors. In adversarial noise scenarios, due to the characteristic of adversarial noise that specifically misleads model decisions, when such noise exists in the input data, it may interfere with the generation quality of pseudo-labels. Especially in the semi-supervised learning stage, incorrect pseudo-labels will be learned and strengthened by the model, leading to the reconstruction result deviating from the true value. Therefore, in future work, we plan to collect image data from real industrial scenarios (such as low-resolution defect images in industrial inspection, blurred images affected by weather in actual monitoring scenarios, etc.) to enhance the model’s robustness in real-world scenarios, where high-fidelity data is often costly or incomplete. We will conduct reconstruction experiments using the SSLMF method to improve the performance of this method in the face of real industrial noise.

In addition, in the current research work, we mainly focus on reconstruction tasks for single-channel or image-like data such as grayscale images and spectrograms, aiming to first consolidate the theoretical and practical foundation of the method in basic image reconstruction scenarios. The selection of these scenarios is based on the need to verify the core mechanism of the method in the early stage of research, so as to observe and analyze the performance of the method more clearly. Extending the method to multi-channel RGB image data is a highly valuable research direction. RGB images contain richer color information and inter-channel correlations. Their reconstruction tasks face new challenges and opportunities in aspects such as feature extraction and channel fusion. Therefore, in our future work, we plan to conduct experiments specifically on multi-channel RGB images, on the one hand, to explore the adaptability of the method in processing color information from different channels and analyze the impact of feature interaction between channels on the reconstruction effect, and on the other hand, in combination with actual application scenarios, to verify the performance of the method with complex color data.

In conclusion, the SSLMF method provides an innovative and effective solution for high-resolution reconstruction in data-scarce scenarios, offering valuable insights into addressing information loss and sample bias through multi-fidelity fusion and semi-supervised learning. Its broad application potential and practical significance make it a promising direction for future research and development.

## Figures and Tables

**Table 1:** Sampling partitions of the nonlinear continuous function.

Data Fidelity	Labeled Sample Size	Unlabeled Sample Size
Low-fidelity	10	10
Medium-fidelity	10	10
High-fidelity	10	0

**Table 2:** Error metric variation with decreasing labeled high-fidelity sample size.

High-Fidelity Labeled Sample Size	Mean Square Error	
	SSLMF	MFDNN
9	**0.0056**	0.0158
8	**0.0055**	0.0325
7	**0.0068**	0.1327
6	**0.0179**	0.1503
5	**0.1223**	0.1596

**Table 3:** Method evaluation metrics.

Methods	PSNR (dB)	SSIM
Proposed method	33.58	0.9363
Benchmark method	26.50	0.9066
RCAN method	31.24	0.9251

**Table 4:** The PSNR(dB) of the models at different learning rates.

Learning Rate	Proposed Method	Benchmark Method	RCAN Method
0.01	27.31	20.08	25.18
0.005	28.87	21.45	25.62
0.001	31.06	22.25	28.47
0.0005	33.01	25.43	30.68
0.0001	33.58	26.50	31.24
**Variance**	5.71	5.90	6.24

**Table 5:** The result of audio spectrum reconstruction.

Methods	PSNR (dB)	SSIM
Proposed method	**31.28**	**0.9011**
R-CED	28.92	0.8591

**Table 6:** Reconstruction performance variance across 10 independent runs on the audio task.

Repetitions	Proposed Method		R-CED	
	PSNR (dB)	SSIM	PSNR (dB)	SSIM
1	31.28	0.9011	28.92	0.8591
2	31.32	0.9164	29.05	0.8608
3	31.10	0.89	27.31	0.7652
4	31.30	0.9021	27.10	0.7312
5	30.98	0.8901	29.01	0.8598
6	31.02	0.8909	26.76	0.6932
7	30.86	0.8805	27.59	0.7532
8	31.35	0.9103	26.03	0.6755
9	31.21	0.9005	26.52	0.6879
10	30.79	0.8636	27.69	0.7829

## Data Availability

The data that support the fundings of this study are available from the corresponding author upon reasonable request.
